# Supplementary material for: Vaccination Strategies against Seasonal Influenza in Long Term Care Setting: Lessons from a Mathematical Modelling Study
Source: Vaccines (Basel). 2022 Dec 23;11(1):32. doi: 10.3390/vaccines11010032 (PMC9861048; doi:10.3390/vaccines11010032)
Supplement: Supplementary file 1 [file vaccines-11-00032-s001.zip › vaccines-2085570-supplementary.pdf]

Vaccination Strategies against Seasonal Influenza in Long Term Care Setting: Lessons from a Mathematical Modelling Study

Supplementary Materials

Matteo Ratti[1], Diego Concina[1], Maurizio Rinaldi[3], Ernesto Salinelli[1], Agnese Maria Di Brisco[2], Daniela Ferrante[1], Alessandro Volpe[1] and Massimiliano Panella[1]

[1] Department of Translational Medicine, University of Piemonte Orientale, Novara, Italy
[2] Department of Economic and Business Studies, University of Piemonte Orientale, Novara, Italy
[3] Department of Pharmaceutical Sciences, University of Piemonte Orientale, Novara, Italy

December 22, 2022

Contents

1 Model: general characteristics **2**
1.1 Introduction . . . 2
1.2 Population model . . . 2
1.3 Population data . . . 4

2 Modeling the influenza spread in the nursing home **5**
2.1 Workers . . . 5
2.1.1 Health care workers (HCW) . . . 6
2.1.2 Nurses (NUR) . . . 8
2.1.3 Physiotherapists, psychologist, animator (OTH) . . . 8
2.2 Guests . . . 9
2.3 Vaccination . . . 10
2.4 Visitors . . . 11
2.5 Contacts . . . 12
2.6 The spreading of influenza . . . 15

3 Simulations and results **17**
3.1 Simulation plan . . . 17
3.2 Comparing real and simulated data . . . 17

4 References **27**

1 Model: general characteristics

1.1 Introduction

We introduce a new model to describe the spreading of influenza in a long-term care nursing home by extending the one introduced in [1] in several aspects and we study its fitting capabilities by using data of a North-Italy structure during the influenza season 2019/2020. Our model is structured with a classical seir (susceptible, exposed, infected and removed) deterministic model describing the influenza spreading in the population and a stochastic agent based model that formalizes the evolution of the disease inside the nursing home. The population model is time continuous, whereas for the stochastic model referred to the nursing home we adopt a discrete-time approach, by setting the time step to 8 hours consistently with the three shifts in the structure: morning shift M (6am-2pm), afternoon shift A (2pm-10pm) and night shift N (10pm-6am).

1.2 Population model

We consider a classical time-continuous seir model, where s is the proportion of susceptible, e the proportion of exposed, i the proportion of infected and r the proportion of removed, so $s+e+i+r=1$. Following [7], we divided the population in to three different age classes:

$$\begin{array}{ccc} 1 = \text{children} & 2 = \text{adult} & 3 = \text{elderly} \\ [0,14] & [15,64] & [65,\omega] \end{array}$$

for each of which there are the same state variables: $s_k,\ e_k,\ i_k,\ r_k,\ k=1,2,3.$
Given the short time window considered, we assume no demography (births, deaths and immigration) and no transitions from one age group to another, so population and its age structure are constant over time. The population dynamic is formalized by the following ordinary differential equations system:

$$\begin{aligned} s_j'(t) &= -\lambda_j\,(t)\,s\,(t) \\ e_j'(t) &= \lambda_j\,(t)\,s_j\,(t) - \sigma e_j\,(t) \\ i_j'(t) &= \sigma e_j\,(t) - \gamma i_j\,(t) \\ r_j'(t) &= \gamma i_j\,(t) \end{aligned}$$

with initial data $s_j(0)$, $e_j(0)$, $i_j(0)$, $r_j(0)$ and $j=1,2,3$.
The infectivity rate σ and the recovery rate γ are assumed the same in all the age classes. The transmission rates β_{kj} depend on age, hence the forces of infection are

$$\lambda_k\,(t) = \sum_j \lambda_{kj}\,(t) \tag{1}$$

where $\lambda_{kj}\,(t) = \beta_{kj} i_j(t)$.
In order to estimate the previous parameters and initial conditions, we use an iterative search based on a least squares principle. We randomly select $N = 2\times10^6$ different parameter configurations in a sufficiently "large" space. For each configuration of parameters, we compute the corresponding simulated weekly incidences and the differences with the real ones. We sum all the square of those differences only for all "central" weeks (from 12th to 28th) and all age classes, obtaining a global least square error. We determine a new (possibly reduced) parameter space based on the best 100 estimations and iterate previous process. We adopt as stop rule a negative variation of the best square error less of 1/100. Figure S1 shows the fitting curves for the three age classes, while Tables S1 and S2 collect the estimated parameters and initial conditions we used in our model.

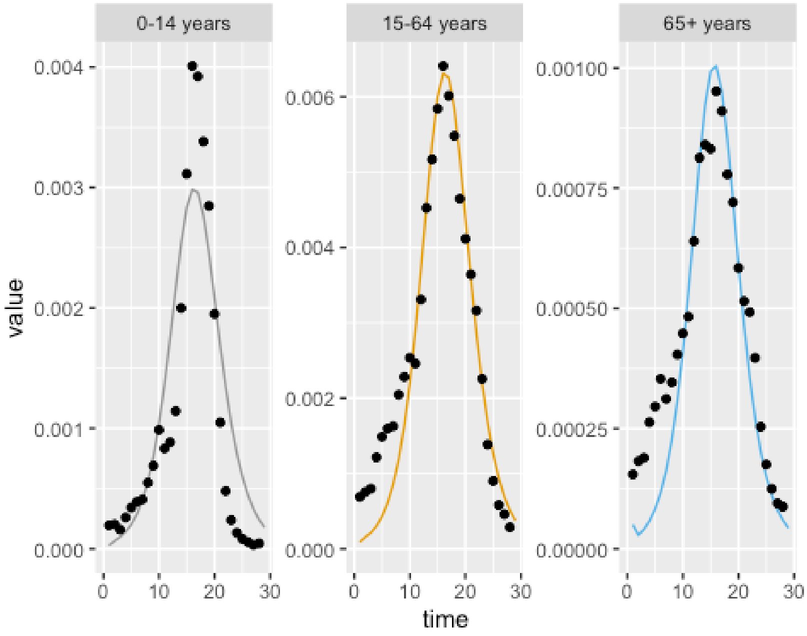

Figure S1: Real (dots) and simulated (line) weekly incidences.

Table S1: Parameters estimates for the population model (shift^{-1}).

parameters	$[0, 14]$	$[15, 65]$	$[65, \omega]$
β_{jk}	β_{11} =0.3007	β_{12} =0.4912	β_{13} =0.4782
	β_{21} =0.3419	β_{22} =0.1166	β_{23} =0.6053
	β_{31} =0.4503	β_{32} =0.9396	β_{33} =0.8956
σ	0.1547	0.1547	0.1547
γ	0.0582	0.0582	0.0582

Table S2: Initial conditions of the population model.

Initial conditions	$[0, 14]$	$[15, 65]$	$[65, \omega]$
$s_j(0)$	$s_1(0)$ =0.068929154	$s_2(0)$ =0.158005673	$s_3(0)$ =0.016085227
$e_j(0)$	$e_1(0)$ =0.00000512	$e_2(0)$ =0.00000032	$e_3(0)$ =0.000041
$i_j(0)$	$i_1(0)$ =0.00000512	$i_2(0)$ =0.00000128	$i_3(0)$ =0.0000205
$r_j(0)$	$r_1(0)$ =0.061060606	$r_2(0)$ =0.480992727	$r_3(0)$ =0.214853333

1.3 Population data

The population of Biella, the province where the LTC facility is located, at the beginning of season is derived from the national statistic institute [6] and summarized in Table S3.

Table S3: Population of the Biella province.

age class	$[0, 14]$	$[15, 65]$	$[65, \omega]$	total
number of individuals	18,926	105,981	50,678	175,585

We consider the influenza season from 42th week of 2019 to the 17th week of 2020 (196 days) because this is the period during which the ministerial surveillance program (INFLUNET) is active and a weekly report about the influenza epidemic is provided by the health authorities [5]. Due to the pandemic situation of the beginning of 2020, the nursing home was closed to visitors after 133 days from the beginning of influenza season (2020/02/24). No others restrictions (like masks, distancing and so on) are considered in the model since they were not really effective in the first months of the pandemic (moreover, there was a scarcity of such measures at the beginning of the pandemic). From INFLUNET reports, the first cases of influenza were registered in the 44th week of 2019 (28/10-3/11); in Piedmont the weekly incidence through the season ranged from < 2.96 to 13.88 per 1.000 individuals. The epidemic peak in Italy was registered during the 5th week of 2020 (27/01-2/02) with a maximum incidence of 13.2 cases per 1.000 individuals; in Piedmont the peak was reached in the 7th week of 2020 (10/2-16/02) with a incidence 11.50 per 1.000 individuals. The estimated total number of the infected in Italy during the 2019/2020 season is 7,595,000 with an attack rate of around 20% [12].

We suppose that a percentage of the population is vaccinated once at the beginning of the season (time 0). For each different age class j, $j = 1, 2, 3$, we consider the vaccine uptake u_j and the vaccine efficacy ve_j. Their values, reported in Table S4, were obtained respectively from the Italian ministry of Health [4] and a recent systematic review of test-negative design studies about influenza vaccine efficacy [9].

Table S4: Vaccine uptake and efficacy for age classes.

age class	$[0, 14]$	$[15, 65]$	$[65, \omega]$
vaccine uptake	$u_1 = 0.0112$	$u_2 = 0.0516$	$u_3 = 0.51$
vaccine efficacy	$ve_1 = 0.4544$	$ve_2 = 0.3744$	$ve_3 = 0.2$

2 Modeling the influenza spread in the nursing home

The considered nursing home is the "Istituto Belletti Bona". The nursing home is located in the centre of Biella (Piedmont, Italy) and belongs to "Cooperativa Sociale Gruppo Anteo". This Long-Term Care service is authorized for 120 beds and subdivided into 4 wards. In this work the four wards are labelled as w_1, w_2, w_3 and w_4, while in the real situation those wards are called with the name of some flowers that begins with the first letters of the alphabet: Anemone (A - w_1), Bucaneve (B - w_2), Camelia (C - w_3) and Dalia (D - w_4). Wards w_1 and w_2 have 20 beds, while w_3 and w_4 have 40 beds. Each room has two beds: changes of rooms rarely happen, so we ignored them in our model.

Guests are allocated to one of the wards on the basis of their activity of daily living (ADL) score measured using the Barthel Score Index [8], [3] and the presence or absence of cognitive impairment. Ward w_1 is dedicated to elderly people with lower levels of dependence in the ADL and with no or mild cognitive impairment. Ward w_2 is dedicated to people with diagnosis of Neurocognitive Disorders with behavioral disorder (such as Alzheimer disease with or without wandering) independently of their level of dependence in the ADL. Guests in ward w_2 usually have a SPSMQ (Short Portable Mental Status Questionnaire) [10] score that defines a severe cognitive impairment. Wards w_3 and w_4 are dedicated to guests with the highest load of care due to the presence of disability consequent to the typical comorbidities of elderly people (such as cardiovascular disease, diabetes mellitus, chronic obstructive disease, stroke, sarcopenia, cancer...).

Each room has two beds and a bathroom; in wards w_1 and w_2 the bathroom is shared with the guest of the same room, while in wards w_3 and w_4 the bathroom is shared between 2 rooms. Each ward has an alarmed entry/exit door that could be opened typing a code number. Each ward has common rooms such as a dining room, a television room and a lounge area that is used for recreational activities in presence of a socio-cultural animator. Physical activity and exercise are usually performed in the presence of a physiotherapist in the ward. Each ward has an assisted bathroom.

A typical day for guests and workers in the nursing home starts at 6.00 AM. In the nursing room of each ward, the morning-shift workers begin their activities with a meet between night-shift workers. Then healthcare workers start their assistance activity (such as hygiene); usually guests are then accompanied to the dining room for the breakfast at 8.00 AM. After breakfast, guests are accompanied by healthcare workers in the common rooms. During the morning at least two guests/day receive a bath in the assisted bathroom healthcare workers. At 10.30 AM usually guests receive hydratation. The lunch is scheduled at 12.00 PM in the dining room. The afternoon shift starts at 2.00 PM. As in the morning in each nursing room of each ward the first activity is the reading and discussion of the clinical records in the presence of the nurse, the psychologist and the physiotherapist. Some guests are placed in bed for the rest. At 4.30 PM guests receive hydratation and a meal in the common room of the ward. Lunch time is at 6.00 PM and guests eat in the dining rooms, at 8.00 PM guests are placed in bed. The night-shift starts at 10 PM with the meet between afternoon-shift workers in the nursing room. The night shift is dedicated to the tidying up of the common areas, cleaning of wheelchairs, and night assistance to guests, with change of absorbent aids and possible hygiene. Absorbent aids are changed at least 4 times/day and when necessary. Pharmacologic therapies are administered by nurses 4 times/day: during the breakfast, during the lunchtime, during the dinner and before guests go to sleep.

2.1 Workers

Workers are divided in the following three categories:

HCW= healthcare worker

NUR= nurse

OTH= others

The last category is made up of different professional figures: two physiotherapists (PHY), a psychologist (PSY) and a socio-cultural animator (ANI). We decided to omit administration staff, cleaners, laundry workers, maintainers or hairdressers having just sporadic contact with guests and workers of the categories

considered. All the workers belong to the adult age class.

Table S5 collects some information about the number and requested presence for each shift, in each ward, for all the different types of workers. Notice that presence differs depending on the considered shift (morning M, afternoon A or night N). The time each worker spends into each ward is equally distributed except for nurses that work more hours in the morning shift. Healthcare workers and nurses are present every day, physiotherapists and the socio-cultural animator work from Monday to Friday, the psychologist is not present on Thursday, Saturday and Sunday.

Table S5: Number and attendance per shift of workers. Numbers between 0 and 1 denote a partial presence in a given ward during a given shift (nurses in wards w_1 and w_2 work for 5 hours in the morning shift and 3 hours in the afternoon shift).

Workers	total number	shift M	shift A	shift N
**HCW**	42	10	8	4
w_1		1	1	1
w_2		2	2	1
w_3		4	3	1
w_4		3	2	1
**Nurses**	8	3	3	0
w_1		0.625	0.375	0
w_2		0.625	0.375	0
w_3		1	1	0
w_4		1	1	0
**Physiotherapists**	2	1	1	0
**Psychologist**	1	0.5	0.5	0
**Animator**	1	0.5	0.5	0

2.1.1 Health care workers (HCW)

Each HCW works around 160 hours per month. There are just a few general rules concerning the assignments of shifts. No more than six consecutive working days and less than 10 night shift per month are possible. We obtained some information about two-months presence of workers in order to estimate the probability of their presence at work. The real number of HCW (more than 50) was reduced in our model eliminating those who have very few presences (less than 3 shift per month). We obtain from the management of the nursing home detailed information about workers shifts in January and March 2020. Based on this information, we conclude that wards w_1 and w_2 share almost all the assigned workers, whereas wards w_3 and w_4 have almost exclusive HCWs. This, probably, implies more cross contacts between the first two wards compared with the others. On the basis of previous information, we obtained frequency tables of each operator per ward which are used in the model to determine the probability that a given operator is present in a given shift, in a given ward (see Tables S6 and S7).

The procedure followed in the model is as follows: for each replica, a distribution of workers for the first shift is drawn at random. The extraction is done by ward, so if a worker does not work in a given ward he can not be extracted. At each shift change, starting from the second one, workers are assigned to wards using the relative frequencies calculated from Tables S6 and S7 as probabilities, respecting the rule that if a worker is present in one shift, he can not be in the next shift.

Table S6: Presence frequency of HCWs in wards w_1 and w_2.

Identifier	M	A	N	ward
1	4	1	5	w_1
2	3	8	0	w_1
3	6	13	12	w_1
4	1	6	16	w_1
5	7	2	1	w_1
12	0	4	6	w_1
23	2	2	3	w_1
25	0	0	3	w_1
34	4	2	3	w_1
37	8	2	0	w_1
38	4	2	5	w_1
39	5	3	0	w_1
40	7	8	4	w_1
41	2	6	4	w_1
42	2	1	0	w_1

Identifier	M	A	N	ward
1	6	9	12	w_2
2	12	4	0	w_2
3	3	4	3	w_2
4	4	6	5	w_2
5	9	13	4	w_2
12	13	10	7	w_2
23	9	14	7	w_2
25	0	0	5	w_2
34	16	10	4	w_2
37	4	4	0	w_2
38	1	15	5	w_2
39	17	11	0	w_2
40	10	10	3	w_2
41	14	11	3	w_2
42	13	5	0	w_2

We assumed that frequency of presences are constant over the simulation period.

Table S7: Presence frequency of HCWs in wards w_3 and w_4.

Identifier	M	A	N	ward
6	20	9	9	w_3
7	14	10	10	w_3
9	9	8	0	w_3
10	31	5	0	w_3
13	28	12	0	w_3
15	4	4	0	w_3
16	8	11	0	w_3
17	23	15	5	w_3
19	0	13	6	w_3
20	4	19	14	w_3
21	17	14	2	w_3
22	32	2	0	w_3
24	2	26	5	w_3
25	0	1	14	w_3
26	31	8	0	w_3
28	15	1	0	w_3
30	12	6	0	w_3
31	10	10	0	w_3

Identifier	M	A	N	ward
8	14	10	10	w_4
9	10	7	2	w_4
11	17	2	0	w_4
14	16	9	2	w_4
15	18	10	0	w_4
16	5	11	0	w_4
18	10	15	6	w_4
25	0	0	2	w_4
27	17	14	2	w_4
28	4	6	0	w_4
29	16	0	3	w_4
30	16	3	0	w_4
31	11	3	3	w_4
32	0	18	15	w_4
33	6	2	0	w_4
35	13	2	0	w_4
36	9	9	14	w_4
37	4	5	1	w_4

2.1.2 Nurses (NUR)

Nurses are 8 and work just in the morning and afternoon shifts. Table S8 collects all their attendance frequencies. In Table S8, notice the introduction of a dummy ward, indicated by $w_{1,2}$. This ward allows to model the fact that some nurses work simultaneously in wards w_1 and w_2 during each of their shifts. Consequently, the attendances recorded in Table S8 are double compared to reality: they are then "weighted" with the information in Table S5.

2.1.3 Physiotherapists, psychologist, animator (OTH)

There are two physiotherapists, one works in the morning, the other one in the afternoon. We assumed that their activity is equally distributed among the four wards (see Table S8). Remember that physiotherapy, like psychological support and animation, is not scheduled on the night shift and the weekend. Table S8 allows to assign a probability of presence to each of the two physiotherapists without additional information. There is only one psychologist available to guests 4 days per week (not present on Thursday, Saturday and Sunday), and one animator present from Monday to Friday. We assumed that their activity is equally distributed among the wards, hence they are present in every ward and shift with a percentage equal to 0.125.

Table S8: Presence frequency of nurses (left) and Physiotherapists (right - from Monday to Friday).

**Identifier**	**M**	**A**	**N**	**Ward**
1	43	43	0	$w_{1,2}$
2	7	3	0	w_3
2	12	12	0	w_4
3	22	19	0	w_3
4	10	11	0	w_3
4	2	4	0	w_4
5	18	23	0	w_3
5	1	0	0	w_4
6	18	20	0	w_4
7	24	17	0	w_2
8	19	19	0	$w_{1,2}$
8	6	5	0	w_3
8	4	10	0	w_4

**Identifier**	**M**	**A**	**N**	**ward**
1	0.25	0	0	w_1
1	0.25	0	0	w_2
1	0.25	0	0	w_3
1	0.25	0	0	w_4
2	0	0.25	0	w_1
2	0	0.25	0	w_2
2	0	0.25	0	w_3
2	0	0.25	0	w_4

Table S9: Flow of workers at each time step, in each ward w_j: probabilities $p_{\text{work}}(t, j, h, k_h)$ are estimated thanks to Tables S6-S8.

**Transition**		**Probability**
at work	$\mathbb{P}\left(W(t+\Delta t, j, h, k_h) = \{1, \cdot\}\right)$	$p_{\text{work}}(t+\Delta t, j, h, k_h)$
at home	$\mathbb{P}\left(W(t+\Delta t, j, h, k_h) = \{0, \cdot\}\right)$	$1 - p_{\text{work}}(t+\Delta t, j, h, k_h)$

We describe the state (see Table S9) of each kind of workers by a two-dimensional variable $W = W(t, j, h, k_h)$ that, depending on time t, ward j, kind h and identifier k_h takes two values: the first concerning the presence

in the nursing home $\{0 = \text{at home}, 1 = \text{at work}\}$, the second one the state of the infection $\{S = \text{susceptible}, E = \text{exposed}, I = \text{infected}, R = \text{removed}\}$. Hence: $W(t, j, h, k_h) \in \{0, 1\} \times \{S, E, I, R\}$, with $t = 1, 2, \ldots, 588$, $j = 1, \ldots, 4$, $h \in \{\text{HCW, NUR, PHY, PSY, ANI}\}$, $k_{\text{HCW}} = 1, 2, \ldots, 42$, $k_{\text{NUR}} = 1, 2, \ldots, 8$, $k_{\text{PHY}} = 1, 2$, $k_{\text{PSY}} = 1$, $k_{\text{ANI}} = 1$.

2.2 Guests

The LTC facility management provided us with data relating to the number, stay, age range and allocation in the different wards of guests during the survey period.
Given the small number of guests in the adult class, in our model we assumed that all belonged to the elderly class. Furthermore, due to the very low number of temporary exits, we assumed that the resignations from the structure were all permanent.

Table S10: General information on the presences of guests. Percentages are computed with respect to the number of available beds.

**# Guests**	w_1	w_2	w_3	w_4
passed through the ward	25 (+25%)	27 (+35%)	63 (+57.5%)	58 (+45%)
always present	13 (65%)	14 (70%)	27 (67.5%)	25 (62.5%)
beds occupied at first	15 (75%)	19 (95%)	37 (92.5%)	36 (90%)
present less than a week	2	0	3	3
present less than two weeks	4	1	7	6

The state of each guest is described by the variable G that, depending on time t, ward j and identifier k takes one of the five possible value vacant V, susceptible S, exposed E, infected I or removed R:

$$G(t, j, k) \in \{V, S, E, I, R\} \qquad t = 1, 2, \ldots, 588,\ j = 1, \ldots, 4,\ k = 1, 2, \ldots, 120.$$

The rate of mortality μ at which a filled bed becomes empty in a given shift and in a given ward (see Table S11), is estimated from the real data: we calculate the sum over time (days from 1 to 196) m_1 of the number of filled beds in a ward and the sum m_2 over time of the number of beds in a ward that become vacant. The rate at which an arbitrary bed of the given ward will become vacant in a given shift is then estimated as $\mu = m_2/(3m_1)$.

Table S11: Guest mortality rates μ_j, $j = 1, 2, 3, 4$.

ward	w_1	w_2	w_3	w_4
μ_j	0.0003813337	0.0003813337	0.0011916679	0.0011916679

Remark 1 *In the population we observed the high number of removed people in the elderly class. This implies a high probability to be removed for elderly people that enter the structure during the influenza season. In some sense, we can expect that a greater turnover in a ward contributes to limit influenza spread. This conclusion appears to be consistent with ARs computed on real data also if it would be important to determine temporally the rate of turnover for each ward during the influenza season.*

For what concerns the mean time of vacancy for a given bed, we assumed that a vacant bed is filled with a probability v_j estimated from real data (see Table S12): for each ward we calculate the number of shifts n_1 when a bed is vacant and the number of shifts n_2 when there is a new access. The probability that a bed vacant at time t will remain vacant at time $t + \Delta t$ is $1 - v_j = 1 - n_2/n_1$.

Table S12: Probability v_j that a vacant bed be filled, $j = 1, 2, 3, 4$.

ward	w_1	w_2	w_3	w_4
v_j	0.003935458	0.003148367	0.009051555	0.008264463

Furthermore we assume that at the beginning of each season (simulation), in each ward, all available beds are occupied and, at any time, the maximum number of occupied beds in a ward cannot exceed the number of available beds in that ward. This last assumption is consistent with the trend in the occupancy rate of the beds at the facility level.
Table S13 collects all previous remarks about flows of guests.

Table S13: Flow of guests at each time step, in each ward w_j.

	**Transition**	**Probability**
mortality	$\mathbb{P}\left(G(t+\Delta t, j, k) = V \mid G(t, j, k) = \neg V\right)$	$\mu_j \Delta t$
susceptible admission	$\mathbb{P}\left(G(t+\Delta t, j, k) = S \mid G(t, j, k) = V\right)$	$s_3(t) v_j \Delta t$
exposed admission	$\mathbb{P}\left(G(t+\Delta t, j, k) = E \mid G(t, j, k) = V\right)$	$e_3(t) v_j \Delta t$
infected admission	$\mathbb{P}\left(G(t+\Delta t, j, k) = I \mid G(t, j, k) = V\right)$	$i_3(t) v_j \Delta t$
removed admission	$\mathbb{P}\left(G(t+\Delta t, j, k) = I \mid G(t, j, k) = V\right)$	$r_3(t) v_j \Delta t$

2.3 Vaccination

Due to the COVID 19 pandemic, it was not possible to obtain detailed and accurate information on workers' vaccination coverage. For this reason, we assumed the same vaccine uptake and efficacy for all the workers categories equal to that of population u_2 and ve_2 (see Table S4).
The information on vaccination with the relative date is not available for all guests. We observed that known vaccination dates were all concentrated at the beginning of our survey (simulation) period and this justifies our assumption that all the vaccination are at time 0. On the basis of the available data, we estimated the data relating to the vaccination coverage of the guests: $u_G = 0.4653$.

Remark 2 *The lack of information on the vaccination only concerns the guests who took over the period. Apart from a few rare exceptions, these people do not get influenza. Some information about their distribution are collected in Tables S14 and S15.*
By previous considerations, we may suppose that "taking over guests" could play a role in mitigating the spread of influenza. Notice that in ward w_1 new entrants are concentrated in the second part of the survey period, so a lower mitigation effect can be expected. This could partly explain the high number of influenza cases recorded in this ward.

Table S14: Distribution per ward of guest with non information about their vaccination (NIV).

ward	w_1	w_2	w_3	w_4
NIV	10	7	23	18
NIV/ #guests	40%	26%	36%	31%

Table S15: Temporal distribution of guests' entrances.

	Oct	Nov	Dec	Jan	Feb	Mar	Apr
w_1	0	0	0	1	4	3	2
w_2	0	2	2	1	0	2	0
w_3	1	5	5	4	5	2	1
w_4	0	3	1	6	3	4	1

The efficacy of the vaccination for guests in the "baseline scenario" is 0.2, equal to that of the population (ve_3 in Table S4).
To measure the effect of the influence on the nursing home, we adopted the usual attack rate, defined for a time interval $[t_1, t_2]$ as:

$$\mathrm{AR}_{[t_1,t_2]} = \frac{\text{number of infected people in the period } [t_1, t_2]}{\text{number of people under risk in the period } [t_1, t_2]}.$$

For the calculation of the attack rate on the real data, we assumed that at time $t = 0$ all guests are susceptible (under risk) and all incoming guests, if not infected, were considered susceptible.
The attack rates for ward and the entire nursing home are collected in Table S16.

Table S16: Estimated real attack rates for wards and facility.

ward	w_1	w_2	w_3	w_4	nursing home
attack rate	0.56	0.3333	0.31746	0.344827	0.36416

2.4 Visitors

Consistently with what happened during the influenza season 2019-2020, we have assumed that visits take place exclusively in the morning and afternoon shifts, and have ceased from day 133 (2020/02/24), since the facility was closed due to the Covid 19 pandemic. All visitors belong to the intermediate age class $15-64$.Even if there was no official visitor register, we chose the most frequent age class as an assumption in our model on the basis of quick interviews of workers. We thought that individual from other age classes acting like visitors may have had contact pattern that closely matched or resembled the one of a middle-age-class individual because it is unlikely that this kind of visitor went unaccompained. In other words, the behavior of individuals belonging to extreme age classes was thought to be similar to the class age 15-64, at least for the time spent inside the nursing home.

We obtain some data on the weekly visits, on the basis of which we obtained the number of average visits per capita and shift shown in Table S17.

Table S17: Number of average visits per capita and shift

w_1	w_2	w_3	w_4
0.04285714	0.04285714	0.06071429	0.0250

Given the great uncertainty about the data and smallness of per capita daily figure (about 0.1), it was decided to multiply these averages by a factor of 4.5.

2.5 Contacts

We distinguish three types of contacts: close [1], characterized by physical contact, casual, with no physical contacts, and "change" which concerns the particular situation relating to the handover between workers at each change of shift. Ten minutes before the shift change, health workers and nurses share the handover in the infirmary of the ward. They discuss the clinical evolution of the host or the specific activities that the guests should participate in. The difficulty of obtaining precise and attentive results when trying to estimate interpersonal contacts is known in the literature (see [11]). To face this problem, contacts in the nursing home were estimated by a survey: staff members were asked to register contacts, distinguishing by type, shift, ward for a week. We calculated an average number of contacts for each type using the data of the survey week and then we normalized with respect to the number of individuals present, obtaining the contacts rates matrices in tables S18-S21.

Table S18: Contact rates in ward w_1

Ward w_1, Morning, Casual	HCW	NUR	GUE	PHY	ANI	PSY
HCW	0	7.857143	3.047619	1.4	1.8	1
NUR		0	2.514286	2.2	0.6	0.75
GUE			0.362585	0.386667	0.213333	0.1
PHY				0	1	0.75
ANI					0	0.75
PSY						0

Ward w_1, Morning, Close	HCW	NUR	GUE	PHY	ANI	PSY
HCW	0	1.714286	2.32381	0.4	0	0
NUR		0	0.485714	0.6	0	0.25
GUE			0.2	0.333333	0.106667	0
PHY				0	0.2	0
ANI					0	0
PSY						0

Ward w_1, Afternoon, Casual	HCW	NUR	GUE	PHY	ANI	PSY
HCW	0	6.285714	4.285714	0.142857	0	1.25
NUR		0	2.314286	0	0	0.5
GUE			0.216327	0.32	0.293333	0.05
PHY				0	0.8	0.5
ANI					0	0.25
PSY						0

Ward w_1, Afternoon, Close	HCW	NUR	GUE	PHY	ANI	PSY
HCW	0	1	4.285714	0	0	0
NUR		0	0.447619	0	0	0
GUE			0.238095	0.306667	0.066667	0
PHY				0	0.2	0
ANI					0	0
PSY						0

Ward w_1, Night, Casual	HCW	NUR	GUE	PHY	ANI	PSY
HCW	0	0	1.428571	0	0	0
NUR		0	0	0	0	0
GUE			0	0	0	0
PHY				0	0	0
ANI					0	0
PSY						0

Ward w_1, Night, Close	HCW	NUR	GUE	PHY	ANI	PSY
HCW	0	0	1.419048	0	0	0
NUR		0	0	0	0	0
GUE			0	0	0	0
PHY				0	0	0
ANI					0	0
PSY						0

Table S19: Contact rates in ward w_2

Ward w2, Morning, Casual	HCW	NUR	GUE	PHY	ANI	PSY
HCW	48.57143	10.28571	4.681818	0.8	0.7	0.375
NUR		0	2.766234	1	1.2	0.5
GUE			0.327273	0.509091	0.345455	0.227273
PHY				0	0.2	0.25
ANI					0	0
PSY						0

Ward w2, Morning, Close	HCW	NUR	GUE	PHY	ANI	PSY
HCW	33.5	3.142857	4	0.3	0	0
NUR		0	2.935065	0.2	0	0
GUE			0.05	2.935065	0.2	0.068182
PHY				0	0	0
ANI					0	0
PSY						0

Ward w2, Afternoon, Casual	HCW	NUR	GUE	PHY	ANI	PSY
HCW	49.42857	5.785714	5.032468	0.2	0	0.25
NUR		0	2.662338	0	0	0.25
GUE			0.163636	0.4	0.163636	0.022727
PHY				0	0	0
ANI					0	0
PSY						0

Ward w2, Afternoon, Close	HCW	NUR	GUE	PHY	ANI	PSY
HCW	33.21429	2.428571	4.850649	0.1	0	0
NUR		0	0.675325	0	0	0
GUE			0.05	0.381818	0.127273	0
PHY				0	0	0
ANI					0	0
PSY						0

Ward w2, Night, Casual	HCW	NUR	GUE	PHY	ANI	PSY
HCW	0	0	2.233766	0	0	0
NUR		0	0	0	0	0
GUE			0	0	0	0
PHY				0	0	0
ANI					0	0
PSY						0

Ward w2, Night, Close	HCW	NUR	GUE	PHY	ANI	PSY
HCW	0	0	2.116883	0	0	0
NUR		0	0	0	0	0
GUE			0	0	0	0
PHY				0	0	0
ANI					0	0
PSY						0

Table S20: Contact rates in ward w_3

Ward w3, Morning, Casual	HCW	NUR	GUE	PHY	ANI	PSY
HCW	19.36905	10.85714	1.98125	2.7	0.75	1.8125
NUR		0	2.082143	3.6	1.6	2.5
GUE			0.095238	0.4	0.215	0.09375
PHY				0	1	1.5
ANI					0	0.75
PSY						0

Ward w3, Morning, Close	HCW	NUR	GUE	PHY	ANI	PSY
HCW	19.70238	7.214286	1.675	0.75	0	0
NUR		0	2.164286	0.8	0	0
GUE			0.034615	0.33	0.07	0.00625
PHY				0	0	0
ANI					0	0
PSY						0

Ward w3, Afternoon, Casual	HCW	NUR	GUE	PHY	ANI	PSY
HCW	27.16667	8.761905	1.897619	1.466667	0.733333	0.25
NUR		0	2.046429	2.8	1.4	0.25
GUE			0.059524	0.41	0.135	0.0375
PHY				0	0.8	0.5
ANI					0	0.25
PSY						0

Ward w3, Afternoon, Close	HCW	NUR	GUE	PHY	ANI	PSY
HCW	27.92857	4.619048	1.984524	0.133333	0.066667	0.083333
NUR		0	2.014286	0.2	0	0
GUE			0.037821	0.37	0.025	0
PHY				0	0	0
ANI					0	0
PSY						0

Ward w3, Night, Casual	HCW	NUR	GUE	PHY	ANI	PSY
HCW	0	0	1	0	0	0
NUR		0	0	0	0	0
GUE			0	0	0	0
PHY				0	0	0
ANI					0	0
PSY						0

Ward w3, Night, Close	HCW	NUR	GUE	PHY	ANI	PSY
HCW	0	0	1	0	0	0
NUR		0	0	0	0	0
GUE			0	0	0	0
PHY				0	0	0
ANI					0	0
PSY						0

Table S21: Contact rates in ward w_4

Ward w_4, Morning, Casual	HCW	NUR	GUE	PHY	ANI	PSY
HCW	31.02381	11.57143	2.288221	2.066667	0.666667	1.5
NUR		0	2.195489	3	0.8	1.5
GUE			0.11644	0.384211	0.173684	0.085526
PHY				0	0.8	1
ANI					0	0.5
PSY						0

Ward w_4, Morning, Close	HCW	NUR	GUE	PHY	ANI	PSY
HCW	28.07143	9.047619	1.972431	1	0.066667	0
NUR		0	1.973684	0.4	0.2	0
GUE			0.052632	0.326316	0.073684	0
PHY				0	0	0
ANI					0	0
PSY						0

Ward w_4, Afternoon, Casual	HCW	NUR	GUE	PHY	ANI	PSY
HCW	93.21429	11.42857	3.167293	1.4	0.9	0.25
NUR		0	2.105263	2.4	0.6	0.5
GUE			0.072445	0.573684	0.121053	0.026316
PHY				0	1.6	0.25
ANI					0	0.25
PSY						0

Ward w_4, Afternoon, Close	HCW	NUR	GUE	PHY	ANI	PSY
HCW	80.85714	1.428571	3.577068	0.1	0	0
NUR		0	1.864662	0.4	0	0
GUE			0.056594	0.378947	0.057895	0
PHY				0	0.2	0
ANI					0	0
PSY						0

Ward w_4, Night, Casual	HCW	NUR	GUE	PHY	ANI	PSY
HCW	0	0	1.879699	0	0	0
NUR		0	0	0	0	0
GUE			0	0	0	0
PHY				0	0	0
ANI					0	0
PSY						0

Ward w_4, Night, Close	HCW	NUR	GUE	PHY	ANI	PSY
HCW	0	0	2.191729	0	0	0
NUR		0	0	0	0	0
GUE			0	0	0	0
PHY				0	0	0
ANI					0	0
PSY						0

Table S22: Contact rates for shift changes

Ward w_1, Change N→M	HCW	NUR
HCW	0.714286	0
NUR		0

Ward w_1, Change M→A	HCW	NUR
HCW	2.785714	0
NUR		0

Ward w_1, Change A→N	HCW	NUR
HCW	1	0
NUR		0

Ward w_2, Change N→M	HCW	NUR
HCW	1.357143	0
NUR		0

Ward w_2, Change M→A	HCW	NUR
HCW	1.285714	0
NUR		0

Ward w_2, Change A→N	HCW	NUR
HCW	0.785714	0
NUR		0

Ward w_3, Change N→M	HCW	NUR
HCW	0.871429	1.128571
NUR	1.128571	0

Ward w_3, Change M→A	HCW	NUR
HCW	1.112245	1.168367
NUR	1.168367	0.5

Ward w_3, Change A→N	HCW	NUR
HCW	0.952381	1.035714
NUR	1.035714	0

Ward w_4, Change N→M	HCW	NUR
HCW	0.976191	1.392857
NUR	1.392857	0

Ward w_4, Change M→A	HCW	NUR
HCW	1.164286	1.271429
NUR	1.271429	1.571429

Ward w_4, Change A→N	HCW	NUR
HCW	0.833333	0.9047619
NUR	0.904762	0

2.6 The spreading of influenza

Let $H = \{\mathrm{GUE}, \mathrm{HCW}, \mathrm{NUR}, \mathrm{PHY}, \mathrm{PSY}, \mathrm{ANI}\}$. Consider the contacts between an individual P of type $h_1 \in H$ and the set $\mathcal{I}_{P,h_2} = \mathcal{I}_{P,h_2}(t)$ of individuals of type $h_2 \in H$ with whom P could have been potentially in contact in a given shift.
Let $K^{\text{casual}}_{h_1,h_2}$ and $K^{\text{close}}_{h_1,h_2}$ be the individual contact rate (in the given shift and ward) of type casual and close respectively between the category of individuals h_1 and h_2.
If the individual P is susceptible and $\mathcal{I}_{P,h_2}$ contains $n^I_{P,h_2}(t)$ infected individuals we model the risk for the first individual of becoming exposed (due to such contacts in the given shift) according to the Reed-Frost equation [13]:

$$\lambda_{P,h_2}(t) = 1 - (1 - p_{\text{casual}})^{n^I_{P,h_2}(t) K^{\text{casual}}_{h_1,h_2} \Delta t} (1 - p_{\text{close}})^{n^I_{P,h_2}(t) K^{\text{close}}_{h_1,h_2} \Delta t}.$$

Notice that $n^I_{P,h_2}(t) K^u_{h_1,h_2} \Delta t$ is just the average number of infected people of type h_2 with whom P had contacts of type u for $u \in \{\text{casual}, \text{close}\}$ at time t.
The risk of infection for P is then given by

$$\lambda_P(t) = 1 - \prod_{h_2 \in H} (1 - \lambda_{P,h_2}(t)).$$

This is the full infection risk on P if the individual P is not a guest. For guests we have additionally to consider the casual contacts with visitors. Let G the selected individual (previously called generically P) when he/she belongs to the guest category. Let $i_2(t)$ be the fraction of infected visitor taken arbitrarily from the model of the 15-64 years population at the given time-step t, and K_{v} the average number of visitors for each turn and ward (remember no visitors at night, see Table S17). Set

$$\omega_{\text{v}}(t) = (1 - p_{\text{casual}})^{i_2(t) K_{\text{v}}}.$$

The total infection risk $\lambda_{G,\text{vis}}$ on the guest G (including the visitor contribution) is then given by

$$\lambda_{G,\text{vis}}(t) = 1 - (1 - \lambda_G(t))\omega_{\text{v}}(t).$$

Notice that at night the contact among guests are reduced to the casual ones among room mates but the previous formulae still hold.

Contacts are regulated now by a single parameter p_{change}. The transmission between workers (hcw and nurses) at each shift change is due to interactions of the workers of the previous shift with the ones and of the current shift. We assume that the change of shift affects just health care workers and nurses. Let $\mathcal{H} = \{HCW, NUR\}$. Consider the contacts between an individual W of type $h_1 \in \mathcal{H}$ and the set $\mathcal{I}_{W,h_2}$ of individuals of type $h_2 \in \mathcal{H}$ with whom the worker W could have been potentially in contact in the given shift change.
Let $K^{\text{change}}_{h_1,h_2}$ be the expected individual contacts at the change of shift between the category of individuals h_1 and h_2.
If the individual W is susceptible and $\mathcal{I}_{W,h_2}$ contains $n^I_{W,h_2}(t)$ infected individuals set

$$\lambda^{\text{change}}_{W,h_2}(t) = 1 - (1 - p_{\text{change}})^{n^I_{W,h_2}(t) K^{\text{change}}_{h_1,h_2}}.$$

The total infection risk of the worker W is then given by

$$\lambda^{\text{change}}_W(t) = 1 - \prod_{h_2 \in H} (1 - \lambda^{\text{change}}_{W,h_2}(t)).$$

The probability that the status of a worker changes form exposed to infected in a given shift is simply given by σ, while the probability that his status changes from infected to removed is given by γ according to the

parameters of the population model (the same probabilities hold when the individual is at work or outside the nursing home).
A change of status of workers form susceptible to exposed outside the nursing home occurs with probability given by the population model (taking in mind that workers belong to the 15-64 years age class).
In the following table G and W, as before, denote a specific guest or worker, t_- and t_+ denote the time before and after the shift change at time t, whereas λ_2 is defined in equation 1.

Table S23: Spreading of influenza in the nursing home.

Workers	**Transition**	**Probability**
from S to E during shift	$\mathbb{P}\left(W(t+\Delta t,j,k)=\{\cdot,E\} \mid W(t,j,k)=\{1,S\}\right)$	$\lambda_W(t)$
from S to E during shift change	$\mathbb{P}(W(t_+,j,k)=\{\cdot,E\} \mid W(t_-,j,k)=\{1,S\})$	$\lambda_W^{\text{change}}(t)$
from S to E (at home)	$\mathbb{P}\left(W(t+\Delta t,j,k)=\{\cdot,E\} \mid W(t,j,k)=\{0,S\}\right)$	$\lambda_2 s_2(t)\Delta t$
from E to I	$\mathbb{P}\left(W(t+\Delta t,j,k)=\{\cdot,I\} \mid W(t,j,k)=\{\cdot,E\}\right)$	$\sigma\Delta t$
from I to R	$\mathbb{P}\left(W(t+\Delta t,j,k)=\{\cdot,R\} \mid W(t,j,k)=\{\cdot,I\}\right)$	$\gamma\Delta t$
Guests	**Transition**	**Probability**
from S to E	$\mathbb{P}\left(G(t+\Delta t,j,k)=E \mid G(t,j,k)=S\right)$	$\lambda_{G,\text{vis}}(t)$
from E to I	$\mathbb{P}\left(G(t+\Delta t,j,k)=I \mid G(t,j,k)=E\right)$	$\sigma\Delta t$
from I to R	$\mathbb{P}\left(G(t+\Delta t,j,k)=R \mid G(t,j,k)=I\right)$	$\gamma\Delta t$

3 Simulations and results

3.1 Simulation plan

We carried out intensive simulation studies (784 scenarios for 200 replications each) with the dual purpose of assessing the fitting ability of the proposed model to real data and examining some relevant public health issues. Therefore, the simulation plan was consistently structured into a baseline scenario and some further explorations. As for the baseline scenario, we explored the fitting ability of the proposed model by letting varying the transmission probabilities (i.e., p_change=0.05, 0.1, 0.15, 0.20, 0.25, 0.30, 0.35, p_casual=(0.15, 0.20, 0.25, 0.30), and p_close=2×p_casual) and keeping constant the remaining parameters. Differently, the further explorations were performed by replicating the baseline scenario for varying levels of vaccine uptake, vaccine efficacy, and percentage of removed at time 0 of workers and guests.

Table S24: Parameters in the baseline scenario.

Symbol	Meaning	Value	Units	Ref
Δt	time step (shift)	8	hours	
T	Duration of simulation	588	shifts	
μ_1	Discharge/mortality rate ward w_1	0.00038	shift^{-1}	estimated
μ_2	Discharge/mortality rate ward w_2	0.00038	shift^{-1}	estimated
μ_3	Discharge/mortality rate ward w_3	0.00119	shift^{-1}	estimated
μ_4	Discharge/mortality rate ward w_4	0.00119	shift^{-1}	estimated
v_1	Change-of-state rate ward w_1	0.00394	shift^{-1}	estimated
v_2	Change-of-state rate ward w_2	0.00315	shift^{-1}	estimated
v_3	Change-of-state rate ward w_3	0.00905	shift^{-1}	estimated
v_4	Change-of-state rate ward w_4	0.00826	shift^{-1}	estimated
σ	Incubation rate	0.1547	shift^{-1}	estimated
γ	Recovery rate	0.0582	shift^{-1}	estimated
$R_W(0)$	Removed fraction of workers at time 0	0.10		[2, 14, 7]
$R_G(0)$	Removed fraction of guests at time 0	0.20		[2, 14, 7]
u_1	Population vaccine uptake, age class 1	0.0112		[4]
u_2	Population vaccine uptake, age class 2	0.0516		[4]
u_3	Population vaccine uptake, age class 3	0.51		[4]
u_G	Guests vaccine uptake	0.4653		estimated
ve_1	Vaccine efficacy age class 1	0.4544		[9]
ve_2	Vaccine efficacy age class 2	0.3744		[9]
ve_3	Vaccine efficacy age class 3	0.2		[9]
p_casual	Transmission probability casual contact	0.15-0.30		
p_change	Transmission probability change contact	0.00-0.35		
ρ	Close/casual transmission probability ratio	2		

3.2 Comparing real and simulated data

For each parameters' configuration, we simulated 200 replicates, obtaining, for each of them, the value of $\text{AR}_{\text{sim},k}$, $k = 1, 2, \ldots, 200$. In order to compare this information with the real AR_{real}, we computed the simulated AR by assuming that all guests who have been in the structure are initially susceptible. In this way, for each simulation, we were able to compute the Root Mean Square Error (RMSE) for a given ward

w_j

$$\mathrm{RMSE}_{T,j} = \sqrt{\frac{1}{200}\sum_{k=1}^{200}(\mathrm{AR}_{\mathrm{sim},T,j,k} - \mathrm{AR}_{\mathrm{real},T,j})^2}$$

or the entire facility:

$$\mathrm{RMSE}_{T} = \sqrt{\frac{1}{200}\sum_{k=1}^{200}(\mathrm{AR}_{\mathrm{sim},T,k} - \mathrm{AR}_{\mathrm{real},T})^2}.$$

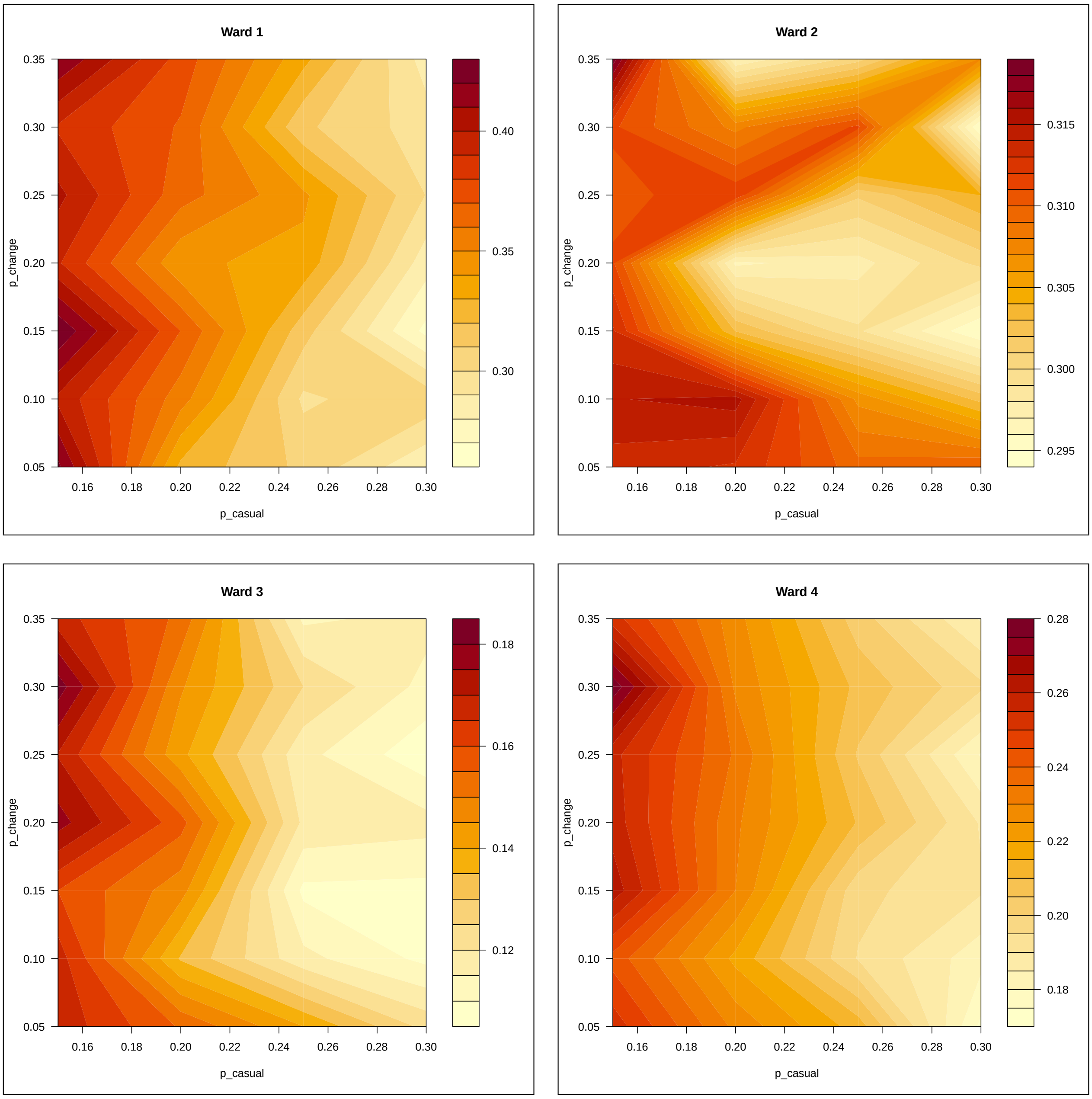

Figure S2: RMSE of ARs for, clockwise from top-left hand panel, wards 1-4.

In Figure S2 the RMSEs referred to the baseline scenario are represented, separately for each ward, as p_casual and p_change vary. In these plots the levels of p_casual are represented on the x-axis, the levels of

p_change are represented on the y-axis, and the plane is colored according to the RMSEs values, from white to dark red as described in the legends on left-hand side of the plots. It is worth noting that in all wards there is a variation in the color gradation from dark red to white along the horizontal axis only. As a result, the empirical evidence suggest that for increasing values of p_casual, the RMSE decreases. Conversely, the parameter concerning the transmission probability p_change appears to have a negligible impact on the RMSEs. The configuration of parameters that minimizes the RMSE computed on the overall nursing home, that is equal to 0.078, is p_casual=0.3 and p_change=0.2. Indeed, this comes with no surprise since the pattern of RMSEs of the nursing home, coherently with what already observed separately for each ward, is strongly affected by the transmission probability p_casual only and the lowest values of RMSEs are observed just in association with the largest value of this parameter.

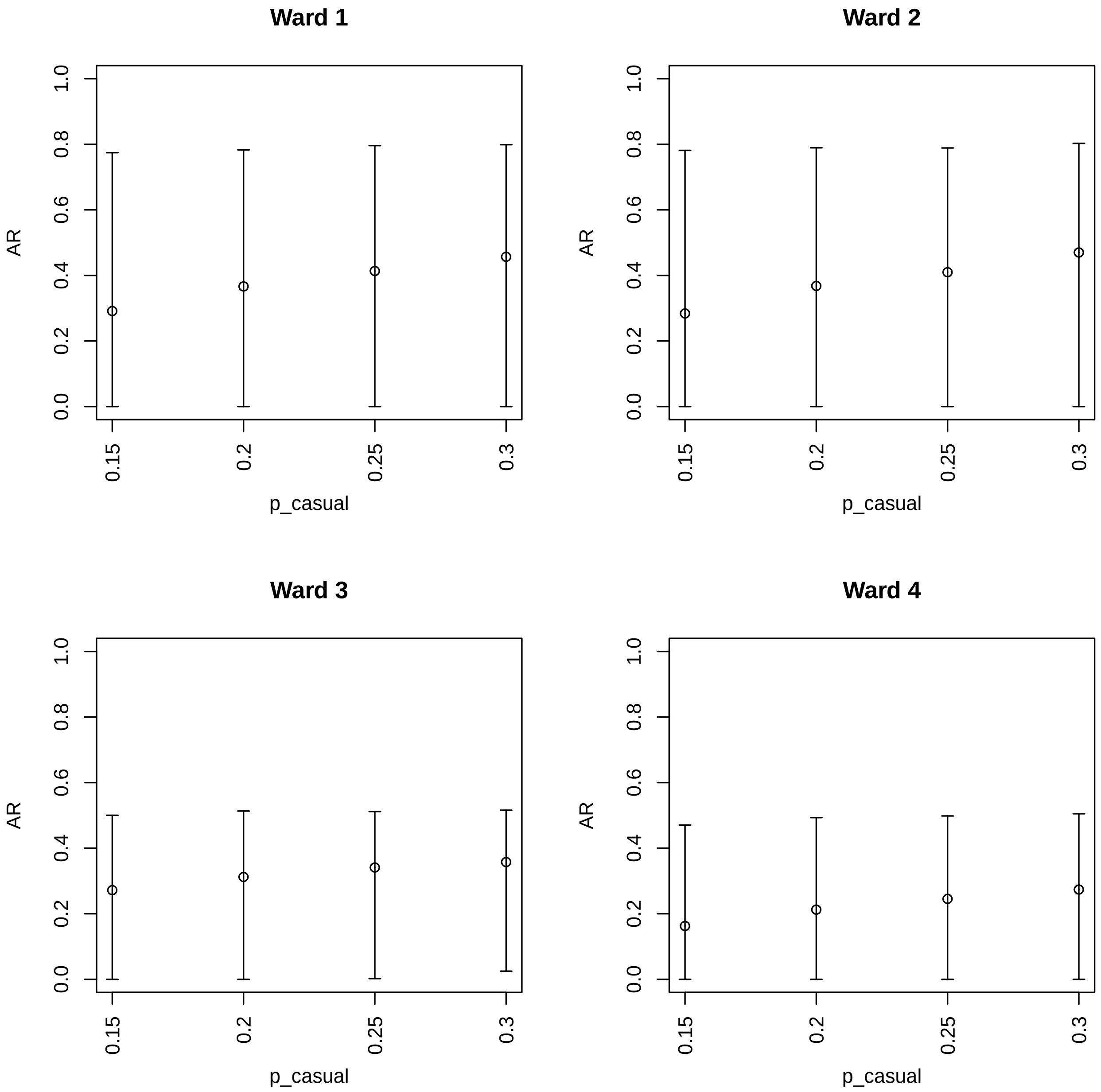

Figure S3: Mean (dots) and 95% CBs (black lines) of AR (present) for the levels of p_casual, for each ward.

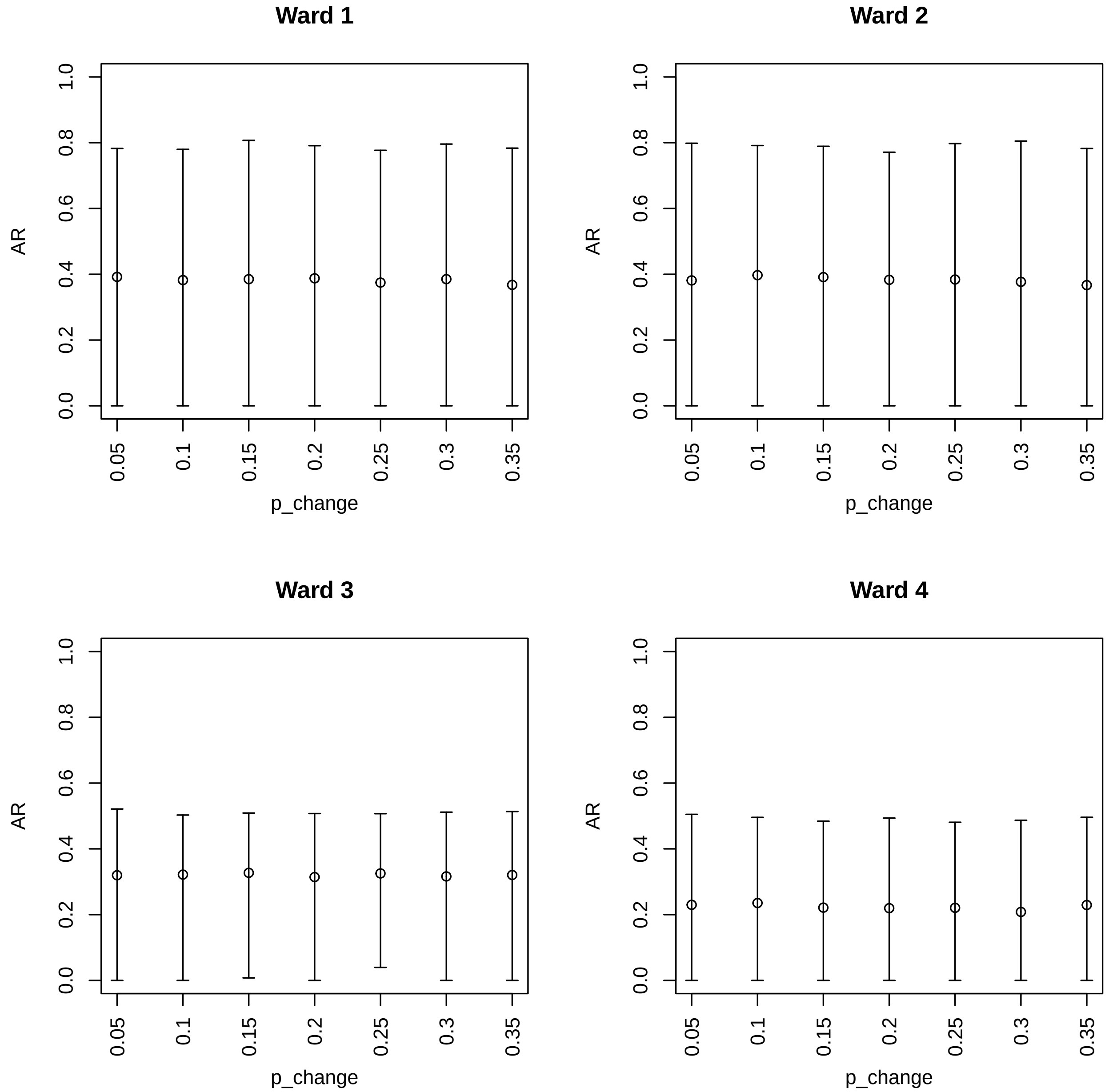

Figure S4: Mean (dots) and 95% CBs (black lines) of AR (present) for the levels of p_change, for each ward.

Further inspecting the baseline scenario, one might be interested in synthesizing the distribution of the AR for varying levels of the transmission probabilities. In Figures S3, S4, and S5 we reported the arithmetic means together with the 95% confidence bounds (CBs), both computed on the Monte Carlo replicates. First, one might observed that results concerning wards 1 and 2 are affected by a greater variability than results concerning wards 3 and 4; this is particularly evident by comparing the range of the 95% CBs that is around 0.8 for the first two wards, and it decreases at around 0.5 for the remaining wards. Despite an exception concerning the CB associated to $\rho = 2$, all remaining CBs have a lower limits of around zero. This result is due to the presence of structural zeros in the simulations results, i.e., a non-negligible fraction of Monte Carlo replicates produced ARs equal to zero. In particular, the fraction of ARs equal to zero is constant under different levels of p_change and of ρ, and it is between 0.2 and 0.3 for wards 1, 2, and 4 whereas it

is slightly less than 0.1 for ward 3. The transmission parameter p_casual has an impact of the fraction of structural zeros: the latter decreases from around 0.5 to around 0.3 as the transmission probability increases in wards 1, 2, and 4. Also in ward 3 the parameter p_casual has a decreasing effect of the fraction of ARs equal to 0, with a variation from around 0.2 to almost 0.

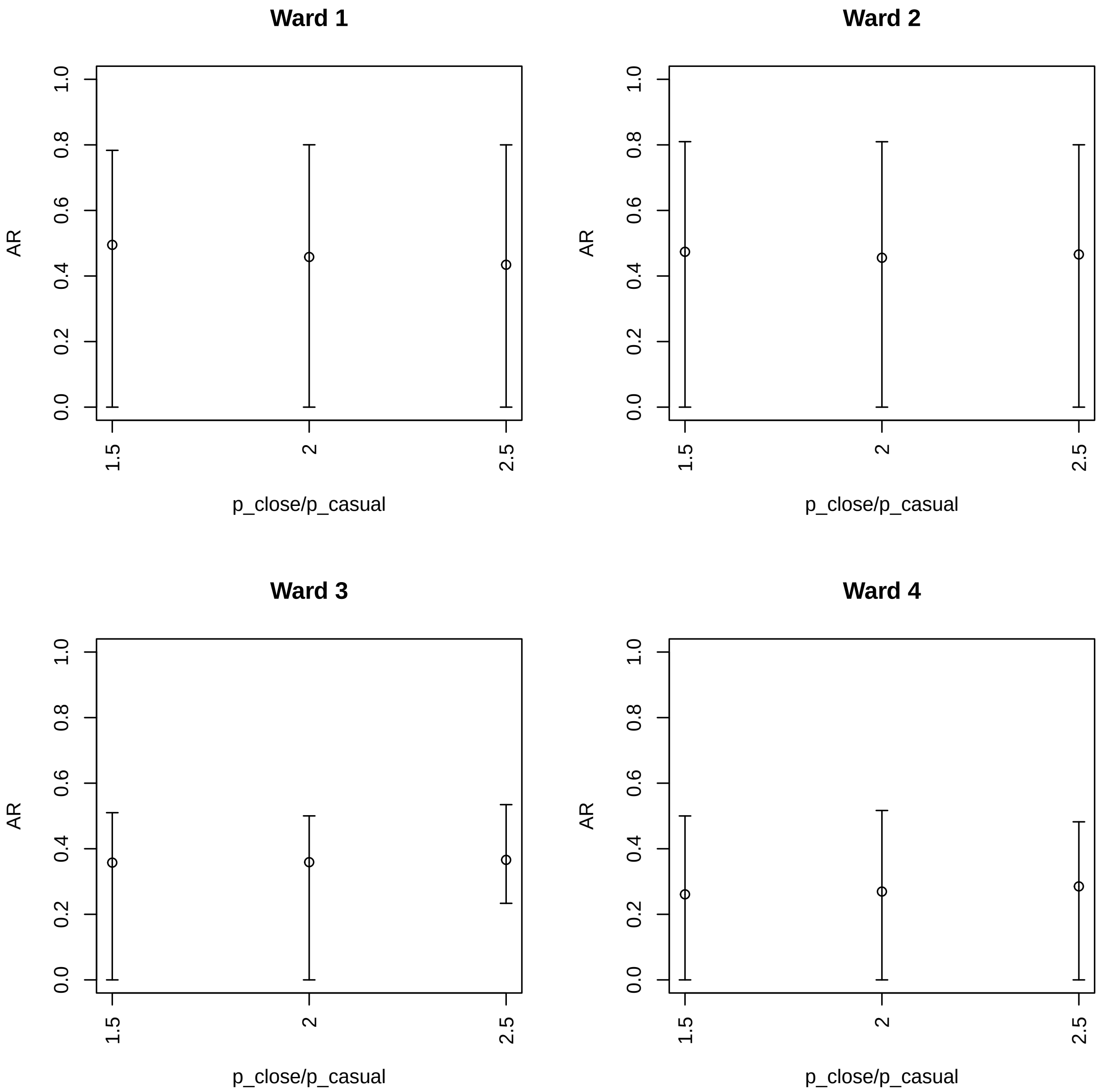

Figure S5: Mean (dots) and 95% CBs (black lines) of AR (present) for the levels of p_close/p_casual, for each ward.

The effect of the parameter p_casual, although not impacting the CBs, appears to be evident in terms of Monte Carlo means. As a result, in Figure S3 it is worth noting that in all wards, and most evidently in the first two, the AR increases for increasing levels of the transmission probability. Results concerning the transmission probability p_change (Figure S4) confirm, instead, the negligible impact of this parameter on the ARs. Indeed, although as many as seven different values of p_change have been explored in simulation studies, the ARs appear to be almost constant within each ward. Finally, by looking at Figure S5 it appears

that the very same considerations as those made for p_change also apply to the close/casual transmission probability ratio.
Moreover, we evaluated the results from some further explorations by inspecting the behaviour of the ARs, separately for each ward, for varying levels of some relevant parameters. Similarly as before, results are synthesized in terms of Monte Carlo means and 95% CBs.
Tables S25 and S26 show results referred to the levels of vaccine uptakes and vaccine efficacy of workers and guests. Neither the vaccine uptake nor the vaccine efficacy seem to affect the ARs' levels. Also results concerning workers and guests seem comparable both in terms of CBs (quite large, especially in the first two wards) and in terms of Monte Carlo means. Interestingly, the most relevant impact is provided by the wards, with ARs that are on average between 0.4 and 0.52 in the first two wards, and between 0.23 and 0.38 in the last two.

Table S25: Mean and 95% CBs (lower bound (LB) and upper bound (UB)) of AR (present) for the levels of vaccine uptake of workers and guests, for each ward.

		Ward 1			Ward 2			Ward 3			Ward 4		
	Levels	Mean	LB	UB	Mean	LB	UB	Mean	LB	UB	Mean	LB	UB
workers	0.00	0.46	0.00	0.80	0.46	0.00	0.80	0.34	0.00	0.50	0.28	0.00	0.50
	0.05	0.46	0.00	0.80	0.46	0.00	0.81	0.36	0.00	0.50	0.27	0.00	0.52
	0.25	0.47	0.00	0.81	0.47	0.00	0.81	0.35	0.00	0.53	0.26	0.00	0.51
	0.50	0.41	0.00	0.76	0.45	0.00	0.76	0.36	0.00	0.56	0.27	0.00	0.48
	0.75	0.47	0.00	0.76	0.49	0.00	0.80	0.36	0.00	0.54	0.26	0.00	0.50
	1.00	0.43	0.00	0.76	0.48	0.00	0.81	0.34	0.00	0.49	0.28	0.00	0.49
guests	0.00	0.52	0.00	0.86	0.53	0.00	0.86	0.38	0.01	0.57	0.31	0.00	0.56
	0.25	0.52	0.00	0.86	0.48	0.00	0.81	0.38	0.24	0.54	0.29	0.00	0.57
	0.46	0.46	0.00	0.80	0.46	0.00	0.81	0.36	0.00	0.50	0.27	0.00	0.52
	0.50	0.44	0.00	0.82	0.47	0.00	0.81	0.36	0.00	0.51	0.27	0.00	0.49
	0.75	0.46	0.00	0.76	0.42	0.00	0.75	0.34	0.01	0.49	0.25	0.00	0.46
	1.00	0.40	0.00	0.73	0.39	0.00	0.76	0.32	0.00	0.52	0.23	0.00	0.43

Table S26: Mean and 95% CBs (lower bound (LB) and upper bound (UB)) of AR (present) for the levels of vaccine efficacy of workers and guests, for each ward.

		Ward 1			Ward 2			Ward 3			Ward 4		
	Levels	Mean	LB	UB	Mean	LB	UB	Mean	LB	UB	Mean	LB	UB
workers	0.10	0.45	0.00	0.77	0.47	0.00	0.81	0.35	0.00	0.53	0.28	0.00	0.51
	0.20	0.46	0.00	0.77	0.46	0.00	0.76	0.37	0.02	0.50	0.28	0.00	0.50
	0.37	0.46	0.00	0.80	0.46	0.00	0.81	0.36	0.00	0.50	0.27	0.00	0.52
	0.50	0.47	0.00	0.80	0.47	0.00	0.85	0.35	0.00	0.50	0.27	0.00	0.52
	0.60	0.48	0.00	0.81	0.48	0.00	0.80	0.34	0.00	0.49	0.29	0.00	0.49
guests	0.10	0.51	0.00	0.85	0.49	0.00	0.85	0.38	0.21	0.56	0.30	0.00	0.53
	0.20	0.46	0.00	0.80	0.46	0.00	0.81	0.36	0.00	0.50	0.27	0.00	0.52
	0.30	0.42	0.00	0.71	0.45	0.00	0.76	0.33	0.00	0.49	0.26	0.00	0.49
	0.40	0.40	0.00	0.80	0.41	0.00	0.70	0.31	0.00	0.48	0.23	0.00	0.47

Figures S6 and S7 show results referred to levels of the fraction of removed at time zero. Focusing on the first Figure, concerning the removed workers at time 0, once again we can observe that as the levels of the parameter vary, both the CBs and the means are quite constant. Similarly as before, the wards seem to have the most significant effects on the ARs. In particular, while in wards 1 and 2 we observe CBs with bounds equal to 0 and 0.8 and an average AR of around 0.5, in wards 3 and 4 the CBs have bounds of 0 and 0.5

and the AR decreases to around 0.3. Conversely, results concerning the removed guests at time 0 suggest an inversely proportional link between the levels of this parameter and the average ARs that is observed in all wards. In particular, the decreasing in the average AR goes from slightly more than 0.6 to around 0.4 in the first two wards, whereas it appears less pronounced in the last two wards where the decrease is from slightly more than 0.4 to around 0.3. Indeed, it emerges that in the first two wards, coherently with what observed so far, the overall ARs are larger than in the last two wards.

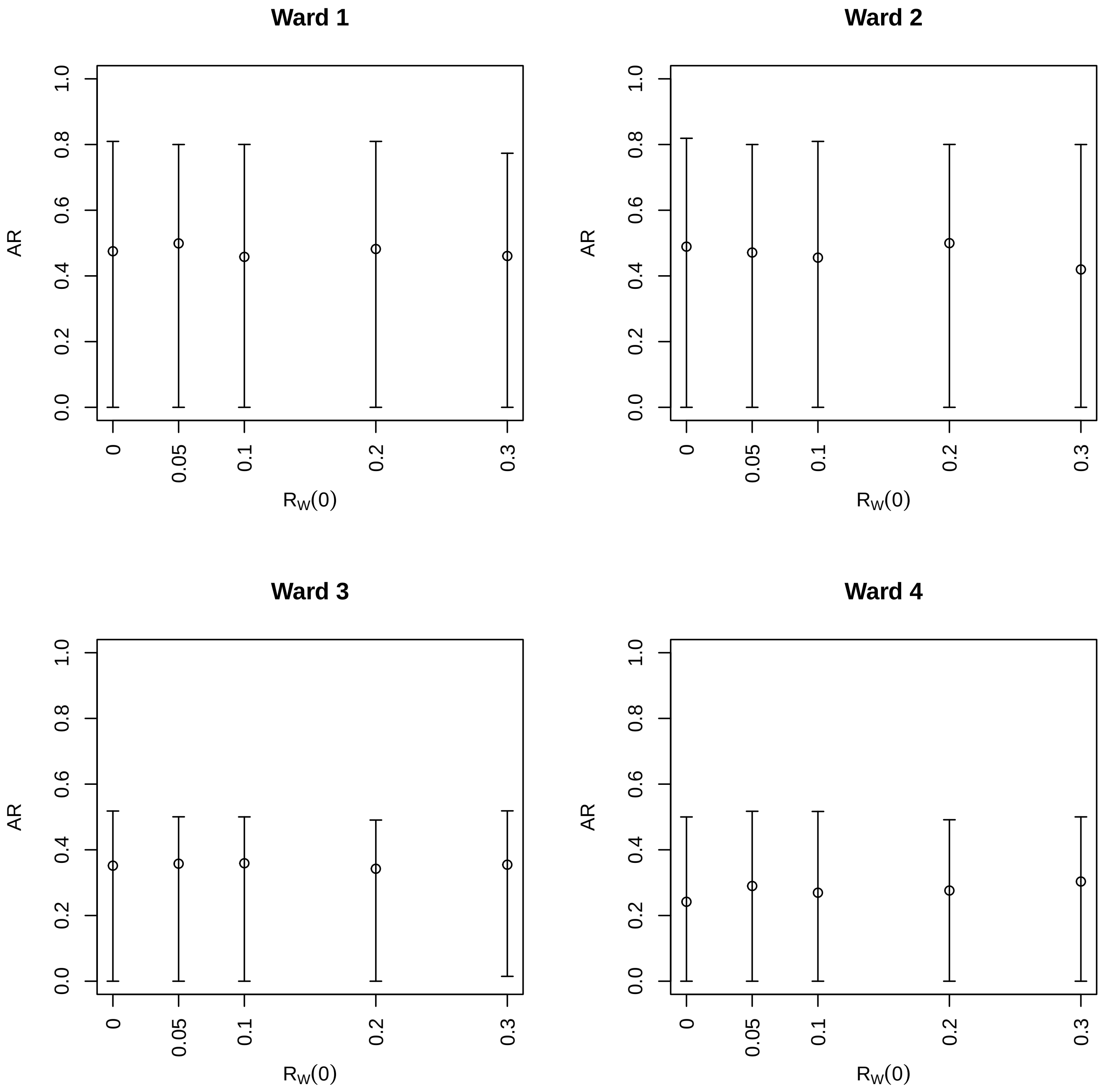

Figure S6: Mean (dots) and 95% CBs (black lines) of AR for the levels of $R_W(0)$, for each ward.

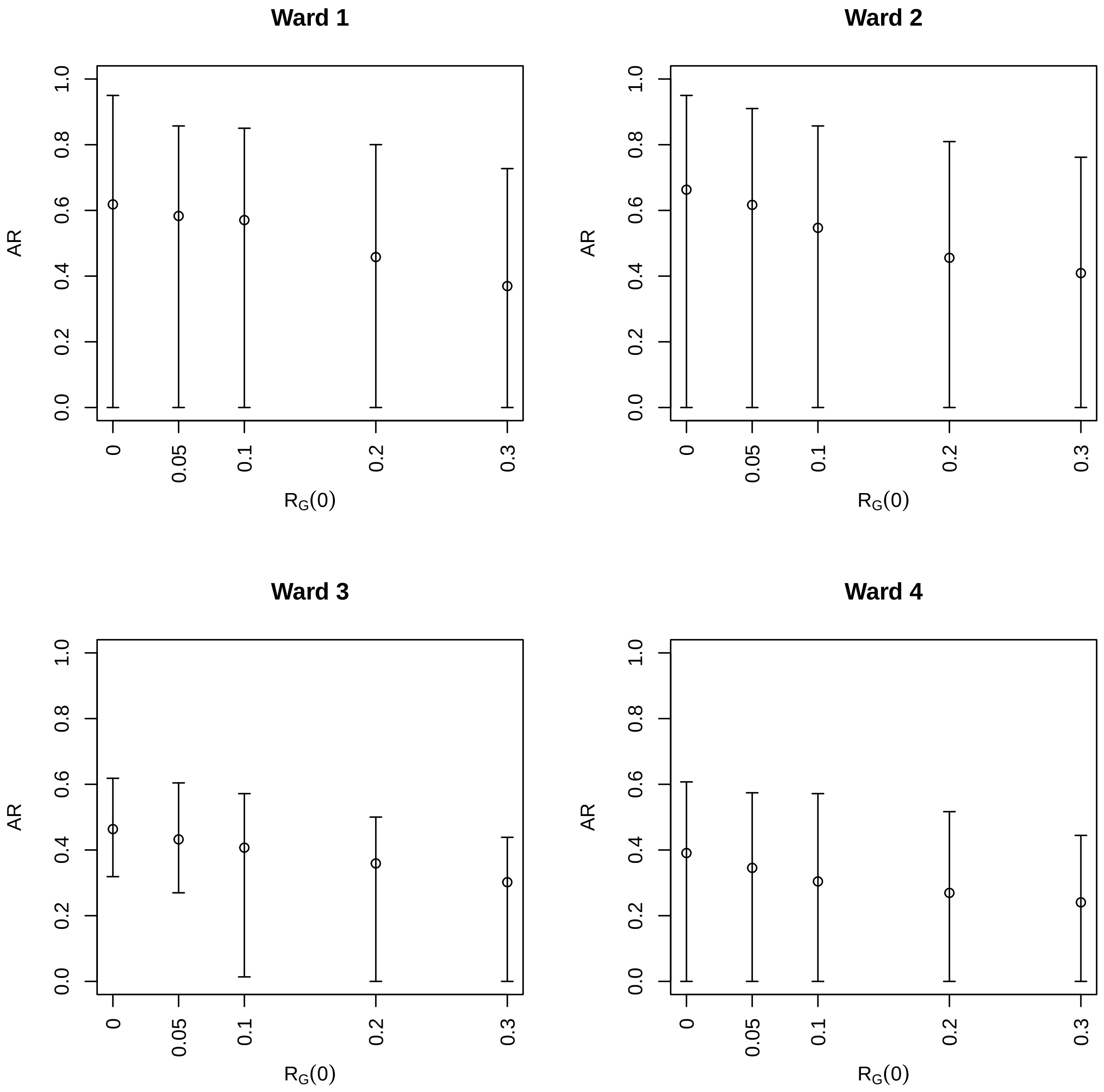

Figure S7: Mean (dots) and 95% CBs (black lines) of AR for the levels of $R_G(0)$, for each ward.

Finally, results referred to varying levels in the percentage of susceptible and in the transmission probabilities, i.e. p_casual and p_change, are illustrated in Figures S8 and S9. It is worth noting that, conditionally on the levels of either p_casual or p_change, the effect of the percentage of initial susceptible is sharp and impacts not only the average ARs but also the 95% confidence bounds. Interestingly, for increasing levels of initial susceptible the average AR increases as well, whereas the associated CBs become more tight. In accordance to what observed so far, once more the first two and last two wards behave, in terms of ARs, comparably.

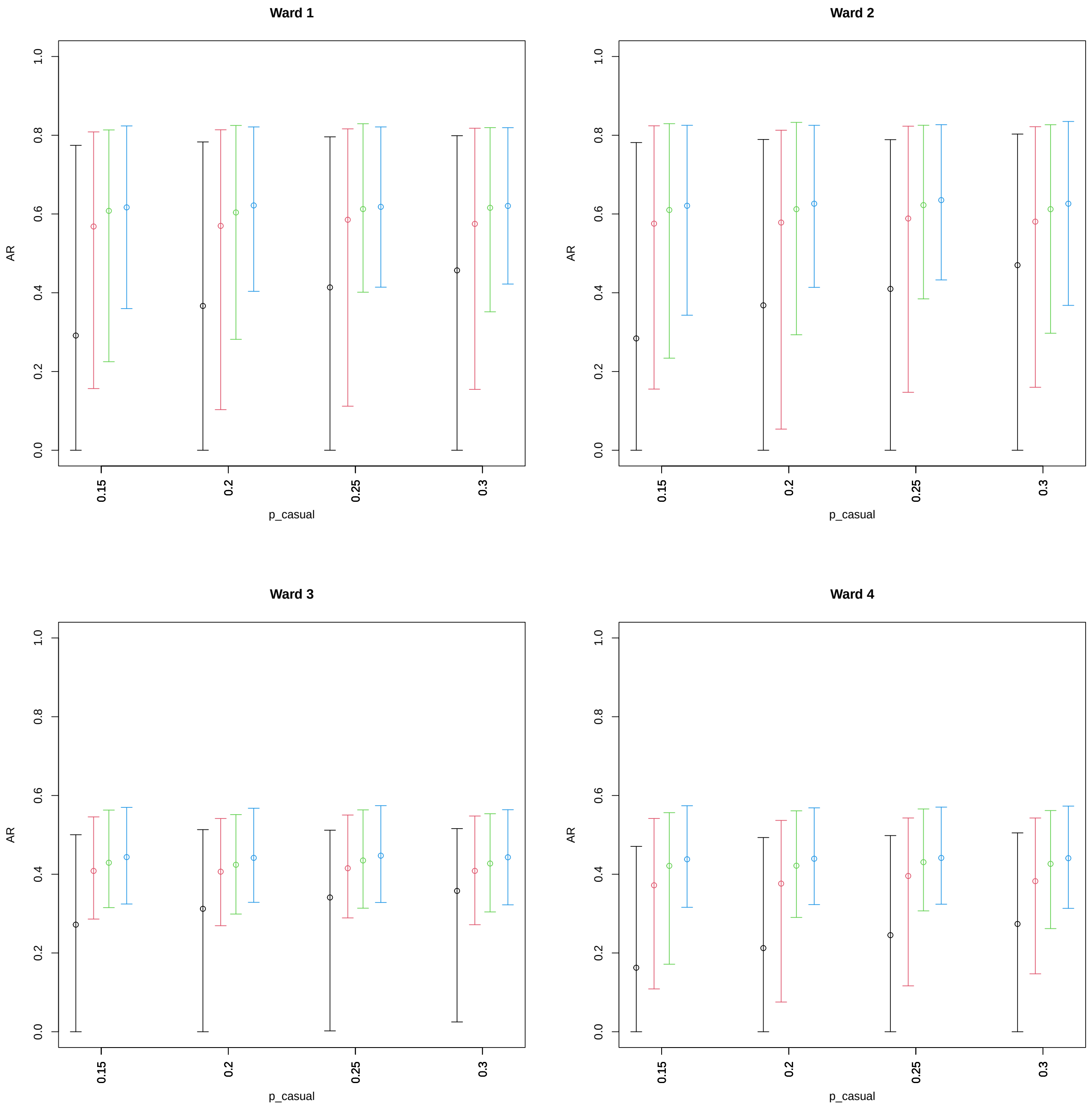

Figure S8: Mean (dots) and 95% CBs (solid lines) of AR for the levels of p_casual, for each ward for baseline scenario (black), Initial Susceptible 31% (red), Initial Susceptible 40% (green), Initial Susceptible 50% (blue).

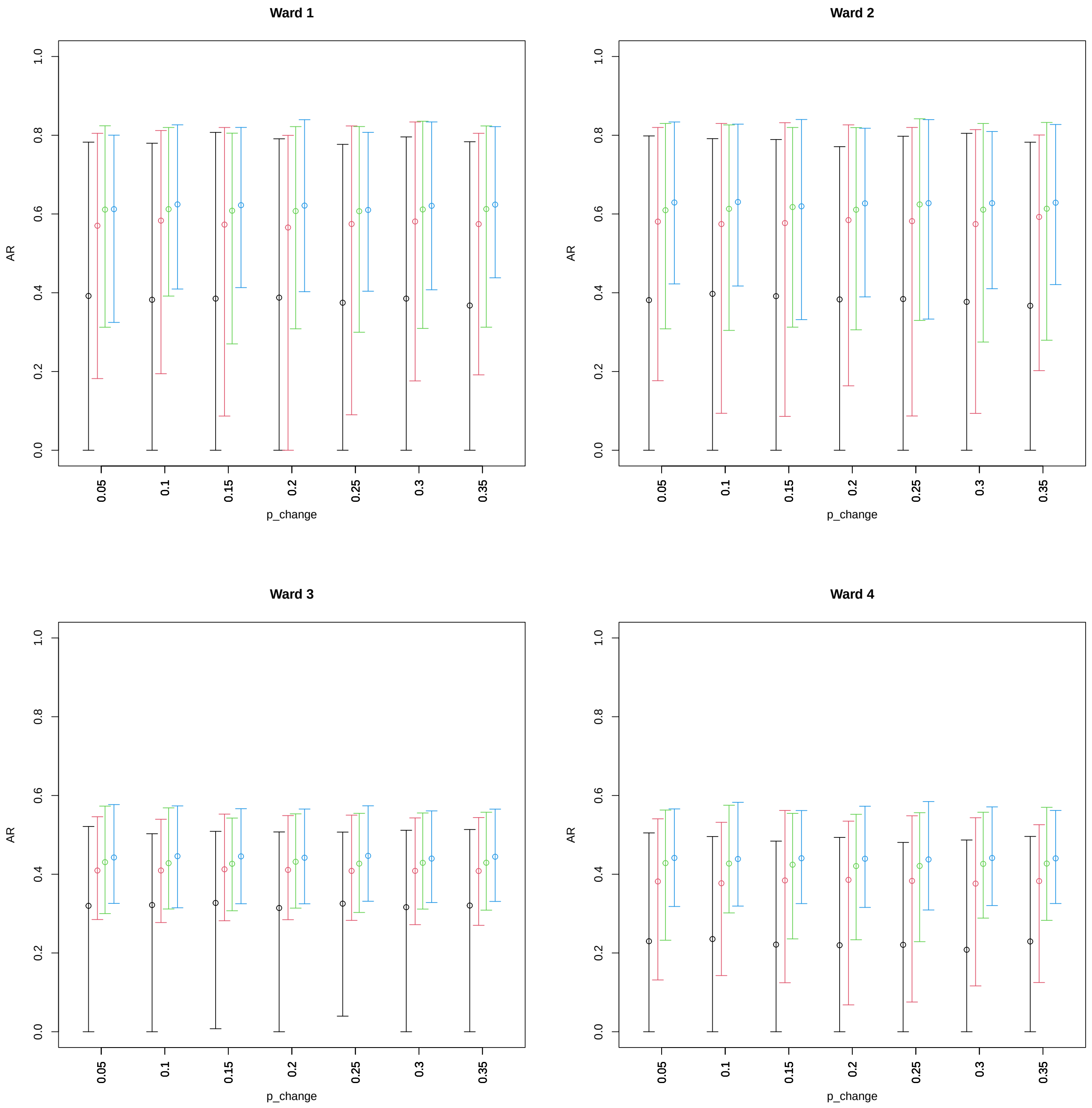

Figure S9: Mean (dots) and 95% CBs (solid lines) of AR for the levels of p_change, for each ward for baseline scenario (black), Initial Susceptible 31% (red), Initial Susceptible 40% (green), Initial Susceptible 50% (blue).

4 References

[1] **van den Dool C., Hak E., Bonten M.J.M., Heijne J.C.M. and Wallinga J.**
The Effects of Influenza Vaccination of Health Care Workers in Nursing Homes: Insights from a Mathematical Model, PLoS Medicine, October 2008, Volume 5, Issue 10

[2] **Dorigatti I. and Cauchemez S. and Pugliese A. and Ferguson N.M.**
A new approach to characterising infectious disease transmission dynamics from sentinel surveillance: application to the Italian 2009-2010 A/H1N1 influenza pandemic. Epidemics, 2012 doi:10.1016/j.epidem.2011.11.001

[3] **Granger, C. V, Dewis, L. S., Peters, N. C., Sherwood, C. C., and Barrett, J. E.**
Stroke rehabilitation: analysis of repeated Barthel index measures. Arch. Phys. Med. Rehabil. 60, 14–7, (1979). Available at: http://www.ncbi.nlm.nih.gov/pubmed/420565

[4] **Health ministerial office**
Vaccinazione antinfluenzale Coperture vaccinali medie (Influenzal Vaccination mean uptakes). URL:https://www.salute.gov.it/portale/documentazione/p6_2_8_3_1.jsp?lingua=italianoid=19 Accessed Aug-08-2022

[5] **INFLUNET Official ministerial influenza surveillance program**
https://www.epicentro.iss.it/influenza/influnet and https://w3.iss.it/site/RMI/influnet/Default.aspx Accessed: May 8th 2021

[6] **ISTAT - Istituto nazionale di statistica**
Italian National Institute of Statistics URL: http://dati.istat.it/Index.aspx?lang=en - accessed: Apr-04-2022

[7] **Lunelli A., Rizzo C., Puzelli S., Bella A., Montomoli E., Rota M.C., Donatelli I., Pugliese A.**
Understanding the dynamics of seasonal influenza in Italy: incidence, transmissibility and population susceptibility in a 9-year period, Influenza and other respiratory viruses, 2012, 286-295

[8] **Mahoney, F. I., and Barthel, D. W** (1965).
Functional Evaluation: The Barthel Index. Md. State Med J. 14, 61–5. Available at: http://www.ncbi.nlm.nih.gov/pubmed/14258950

[9] **Okoli, G. N. and Racovitan, F. and Abdulwahid, T. and Righolt, C. H. and Mahmud, S. M.**
Variable seasonal influenza vaccine effectiveness across geographical regions, age groups and levels of vaccine antigenic similarity with circulating virus strains: A systematic review and meta-analysis of the evidence from test-negative design studies after the 2009/10 influenza pandemic. Vaccine, 2021. doi: 10.1016/j.vaccine.2021.01.032

[10] **Pfeiffer, E.** (1975).
A Short Portable Mental Status Questionnaire for the Assessment of Organic Brain Deficit in Elderly Patients†. J. Am. Geriatr. Soc. 23, 433–441. doi: 10.1111/j.1532-5415.1975.tb00927.x.

[11] **Read J.M., Edmunds W.J., Riley S., Lessler J., Cummings D.A.T.**,
Close encounters of the infectious kind: methods to measure social mixing behaviour. Epidemiol. Infect. 2012, 140, 2117-2130

[12] **Trentini, F.; Pariani, E.; Bella, A.; Diurno, G.; Crottogini, L.; Rizzo, C.; Merler, S.; Ajelli, M.**,

Characterizing the transmission patterns of seasonal influenza in Italy: lessons from the last decade. BMC Public Health 2022, 22, 1–9. https://doi.org/10.1186/s1 2889-021-12426-9.

[13] **Vynnycky, E., White R.G.**
Infectious Disease Modelling, Oxford University Press,(2011).

[14] **Yaari, R. and Katriel, G. and Stone, L. and Mendelson, E. and Mandelboim, M. and Huppert, A.**
Model-based reconstruction of an epidemic using multiple datasets: Understanding influenza A/H1N1 pandemic dynamics in Israel. Journal of the Royal Society Interface, 2016. doi:10.1098/rsif.2016.0099
